# Parental Influence: Childhood caregiver well‐being associates with epigenetic aging among older adults

**DOI:** 10.1002/alz70858_104383

**Published:** 2025-12-26

**Authors:** Shana D. Stites, Morgann Adams, Carolyn Kuz, Kimberly Halberstadter, Valerie Humphreys, Corey T. McMillan, Dawn Mechanic‐Hamilton

**Affiliations:** ^1^ University of Pennsylvania, Philadelphia, PA, USA; ^2^ Temple University, Philadelphia, PA, USA

## Abstract

**Background:**

A parent's well‐being is instrumental for the child's safety, health and enrichment and these factors are all known to contribute to poorer late‐life health outcomes. Epigenetic age, measured using DNA methylation, represents a biological feature of aging that reflects the extent to which an individual is accelerated or decelerated relative to their chronological age. Here we evaluate how parental relationship type and well‐being associate with later life epigenetic age.

**Method:**

Cognitively unimpaired older (>65 years) adults (*N* = 152) completed the Penn Alzheimer's Disease Research Center's Life Experience Survey. Questions inquired about relationship type (i.e., mother, father, aunt etc.) and overall well‐being of up to three childhood caregivers. Ratings were from 0‐100 with higher being better. Participants also completed measures of socioeconomic status and sociodemographic data. DNA methylation was measured using genomic blood and the Illumina EPIC array to compute Horvath and Levine epigenetic clocks. Linear regression models examined effects of parental relationship type and parental well‐being ratings on age acceleration in bivariate models, which permitted examination of single parents and types of dyads (i.e., mother‐father, mother‐grandmother) and trivariate models, which allowed each parent relationship type to covary independently. Multivariable models controlled for potential confounding from socioeconomic status, adverse childhood events, and key demographic factors.

**Results:**

Father relationship type showed a statistical trend toward decelerated aging (Horvath, β=3.5, 95%CI ‐0.02 to 7.0). In bivariate models, better parental well‐being was associated with decelerated aging (Levin, β=0.14, 95%CI 0.02, 0.24). Secondary parent well‐being showed no effect. In trivariate models, better primary parent well‐being was associated with decelerated aging (Levin, β=0.14, 95%CI 0.02, 0.24; Horvath, β=0.09, 95%CI 0.03, 0.10). Secondary parent well‐being higher than the primary parent's was associated with accelerated aging (Levin, β=‐0.08, 95%CI ‐0.2 to 0.02, *p* = 0.05; Horvath, β=‐0.09, 95%CI ‐0.1, ‐0.03). These associations remained in multivariable models.

**Conclusion:**

Our findings provide evidence for protective and negative biological aging outcomes associated with primary compared to secondary parental well‐being. Thus, it is important to consider social context on aging outcomes. The findings underscore the import of caregivers in the health of older adults. This research line may inform population‐level health interventions.

